# Budd-Chiari Syndrome with Multiple Thrombi due to a Familial Arg42Ser Mutation in the Protein C Gene

**DOI:** 10.1155/2013/270419

**Published:** 2013-10-24

**Authors:** Jun Muratsu, Atsuyuki Morishima, Kazuhiro Mizoguchi, Keiji Ataka, Hiroshi Yamamoto, Xinping Fan, Toshiyuki Miyata, Katsuhiko Sakaguchi

**Affiliations:** ^1^Department of Nephrology and Hypertension, Sumitomo Hospital, 5-3-20 Nakanoshima, Kitaku, Osaka 530-0005, Japan; ^2^Department of Cardiovascular Surgery, Sumitomo Hospital, 5-3-20 Nakanoshima, Kitaku, Osaka 530-0005, Japan; ^3^Department of Radiology, Sumitomo Hospital, 5-3-20 Nakanoshima, Kitaku, Osaka 530-0005, Japan; ^4^Department of Molecular Pathogenesis, National Cerebral and Cardiovascular Center, 5-7-1 Fujishirodai, Suita, Osaka 565-8565, Japan

## Abstract

A 34-year-old Japanese woman was admitted to our hospital complaining of developing bilateral pedal edema. Imaging studies led to a diagnosis of Budd-Chiari syndrome combined with internal jugular vein thrombus. We investigated the cause of thrombosis and found that the anticoagulant activity of protein C was decreased. Genetic analysis showed the presence of a c.125C>A (Arg42Ser) substitution in the protein C gene (*PROC*) of the proband, which generates an Arg42Ser mutation that replaces the scissile bond Arg42-Ala43 normally cleaved by a furin-like processing protease. Her father and younger brother also carried this mutation, although they had no evidence of thrombosis.

## 1. Introduction

Budd-Chiari syndrome is defined as any pathophysiologic process, such as thrombosis, that results in an interruption or diminution of the normal flow of blood from the liver [1]. One cause of this syndrome is a deficiency in protein C [2–4], a precursor of a vitamin K-dependent serine protease that plays an important role in the regulation of blood coagulation [5–7]. Heterozygous protein C deficiency is inherited in an autosomal dominant fashion, and hereditary deficiency in this protein is associated with a high risk of thrombotic disease [8]. A c.125C>A change in the protein C gene (*PROC*) is designated protein C Osaka 10. This mutation had been reported in only one case so far [9]. Although some cases of Budd-Chiari syndrome have been reported to be caused by hereditary protein C deficiency, genetic analysis of family members was not investigated. 

To our knowledge, the present study represents the first case report describing Budd-Chiari syndrome combined with multiple thrombotic lesions and genetic analysis of family members.

## 2. Case Report

A 34-year-old Japanese woman was admitted to our hospital complaining of a weight gain of up to 15 kg and increased bilateral pedal edema over the preceding 3 weeks. She did not have a history of these symptoms and was not taking any medications. On admission, her pulse rate was 84 beats/min, her blood pressure was 130/84 mmHg, and her body temperature, 36.7°C. Her consciousness was alert, her bulbar conjunctiva were not icteric, and the palpebral conjunctiva were not pale. No respiratory rales or heart murmurs were noted on auscultation. Palpation revealed hepatosplenomegaly and ascites with no enlargement of the thyroid gland. Neurological abnormalities, jaundice, or palmar erythema was not evident. However, bilateral pedal edema was observed.

**Figure 1:**
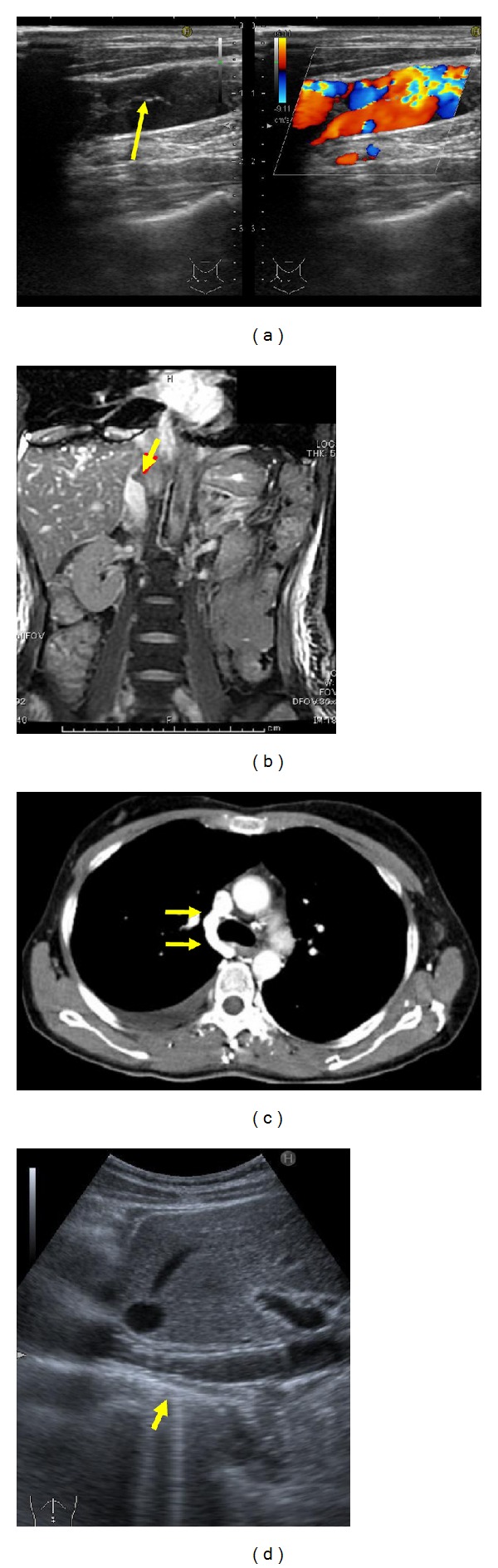
Imaging studies. (a) Carotid ultrasonography shows a thrombus of the right internal jugular vein indicated by the arrow. Blood flow in the right internal jugular vein was maintained. (b) Abdominal MRI first imaging employing steady state acuisition (FIESTA) reveals a high-intensity signal emanating from the intrahepatic portion of the inferior vena cava (arrow), which suggested thrombus. (c) Thoracic CT during injection of contrast medium reveals right pleural effusion and dilatation of an azygos vein at the arrows. (d) Abdominal Doppler ultrasonography shows complete patency (arrow) and no ectopia of the stent. This image was acquired 16 months after the patient was admitted.

**Figure 2:**
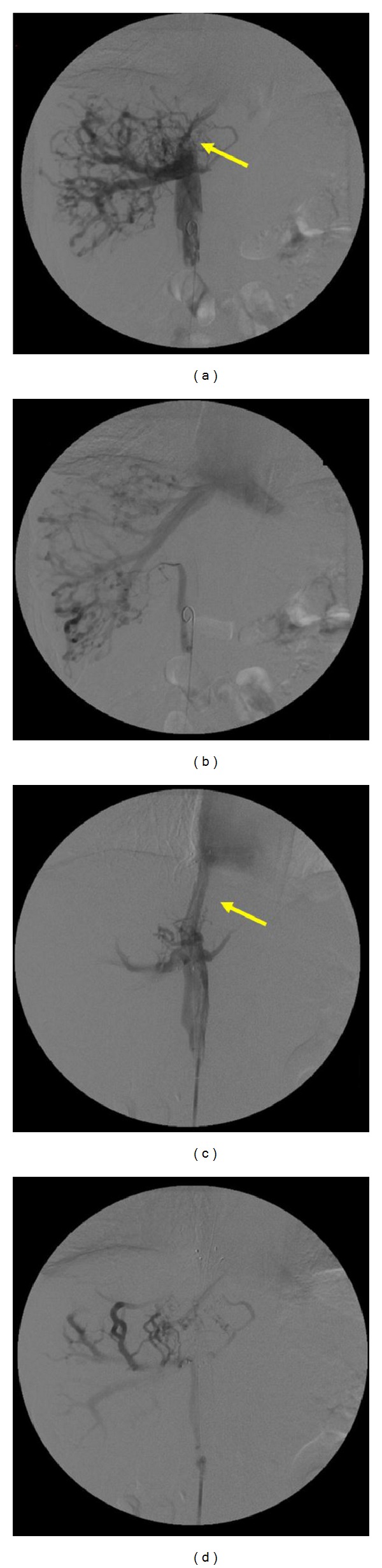
Angiography. ((a), (b)) Angiography reveals a thrombus in the inferior vena cava, which extends from its outlet to the right atrium. The intrahepatic collaterals are also shown. A portion of thrombus is indicated by the arrow. ((c), (d)) We performed PTA with stent implantation (LUMINEXX 14 × 80 mm) and successful balloon dilatation (OPTA Pro 10 × 40 mm) of the inferior vena cava at its outlet to the right atrium. The arrow points to the the stent.

Laboratory data acquired upon admission are shown in Table 1. Alanine aminotransferase (ALT) (46 IU/L) and D-dimer (1.40 *μ*g/mL) were slightly elevated. Her chest X-ray and echocardiogram were normal, but carotid ultrasonography showed a thrombus of the right internal jugular vein (Figure 1(a)). Blood flow in the right internal jugular vein was maintained and did not require immediate treatment. Abdominal ultrasonography and computed tomography (CT) showed hepatomegaly, ascites, gallbladder wall thickening, and a thrombus within the intrahepatic portion of the inferior vena cava. Abdominal magnetic resonance imaging (MRI) revealed a region of high-intensity, which suggested the presence of a thrombus within the inferior vena cava (Figure 1(b)). Abdominal-pelvic CT and MRI analyses did not detect tumors. The presence of right pleural effusion and dilatation of an azygos vein were evident in CT images acquired following the injection of contrast medium (Figure 1(c)). Venous angiography revealed a thrombus within the inferior vena cava at its outlet that extended to the right atrium and was accompanied by intrahepatic collaterals (Figures 2(a) and 2(b)). This patient was therefore diagnosed with Budd-Chiari syndrome.

Based on the results of a previous study, we expected that percutaneous transluminal angioplasty (PTA), a minimally invasive procedure, would result in a favorable outcome [10]. We performed PTA with stent implantation (LUMINEXX 14 × 80 mm) and successful balloon dilatation (OPTA Pro 10 × 40 mm) from the outlet of the inferior vena cava to the right atrium (Figures 2(c) and 2(d)). The pressure of the inferior vena cava decreased after PTA from 25 mmHg to 16 mmHg. Although we prepared the operation for a case of pulmonary embolism, PTA was completed without any complications. We started treatment by administering intravenous heparin sodium (10,000 units/day). Heparin sodium infusion was discontinued 2 days after PTA, and the patient's postoperative course was uneventful. Abdominal CT during injection of contrast medium was performed on postoperative day 10 and showed the patency of the inferior vena cava. Abdominal ultrasonography showed that hepatomegaly, ascites, and gallbladder wall thickening were diminished. Bilateral pedal edema was dramatically alleviated after 14 days.

We examined protein C anticoagulant activity and it was decreased to 44% (normal: 64%–146%), contributing to thrombogenesis. Protein C anticoagulant activity was decreased in her father (61%) and younger brother (62%) (Table 2). While the medical histories of her younger brother and father were not informative, her younger brother had been suffering for several years from idiopathic recurrent chest pain. 

To determine whether or not the patient and her family members harbored *PROC *mutations, we performed genetic analyses after we obtained informed written consent. Genomic DNA was isolated from blood samples using a QIAamp DNA Blood MiniKit kit (Qiagen). All exons and flanking intronic regions of *PROC* were amplified using polymerase chain reaction (PCR) and then sequenced in both sense and antisense strands using an ABI Prism BigDye Terminator v3.1 Cycle Sequencing kit (Applied Biosystems) [11]. Primer sequences are available on request. A c.125C>A change that encodes an Arg42Ser mutation was identified in the patient. Arg42 is located at the cleavage site recognized by the furin-like protease that normally processes preproprotein C to its mature form.

**Figure 3:**
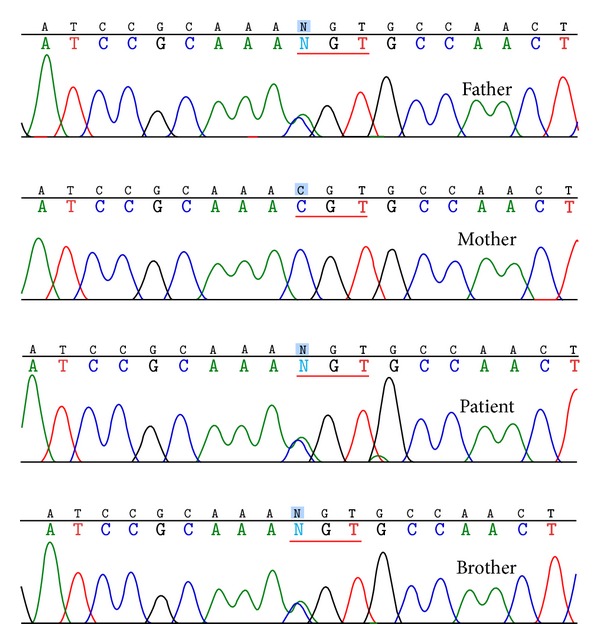
DNA sequence analysis. A c.125C>A substitution in the *PROC* sequence of the proband. The CGT encoding Arg42 at the processing cleavage site, which is recognized by the processing enzyme, is changed to AGT, encoding Ser. This mutation was also identified in her father and her younger brother but not in her mother.

This mutation was also identified in the patient's father and younger brother, whereas the sequence of her mother's DNA in this region was found to be wild-type (Figure 3). Although her younger brother did not have evidence of thrombosis, his idiopathic recurrent chest pain may be shown by pulmonary embolism due to microscopic thrombosis. And he desired administration of warfarin. Warfarin (3–6 mg/day orally) was given to the patient and younger brother according to the levels of prothrombin time-international normalized ratio (maintaining between 2.0 and 2.5). Abdominal Doppler ultrasonography, which was performed during a follow-up visit to the outpatient clinic 16 months after the procedure, showed complete patency and no ectopia of the stent (Figure 1(d)). Carotid ultrasonography showed that blood flow in the right internal jugular vein was maintained. 

## 3. Discussion

 We describe here a patient with Budd-Chiari syndrome with multiple venous thrombi caused by an Arg42Ser mutation in *PROC*. Her father and younger brother also carried this mutation; however, they were free of detectable thrombosis. Her mother was genetically unaffected. 

Budd-Chiari syndrome is defined as any pathophysiologic process that results in an interruption or diminution of the normal flow of blood out of the liver. This presentation is uncommon, often dramatic illness characterized by abdominal pain, ascites, hepatomegaly and a poor prognosis [1]. The diagnosis can be established noninvasively by ultrasonography with Doppler studies, CT scan, or magnetic resonance angiography (MRI angiography) [12–14]. Treatment depends upon the cause, the anatomic location, and extent of the thrombotic process [15]. The etiology of Budd-Chiari syndrome, a rare disorder, is varied, and its cause remains unknown in most patients [16]. Analysis of 157 patients with Budd-Chiari syndrome in Japan revealed that 141 were idiopathic, 2 were deficient in protein C, 2 were deficient in protein S, and 1 was deficient in antithrombin [17]. Protein C deficiency is one of the causes of Budd-Chiari syndrome [2–4]. 

Protein C is the precursor of a vitamin K-dependent serine protease that plays an important role in the regulation of blood coagulation [5–7]. The observed molecular mass of protein C is approximately 62 kDa, accounted for by one light chain (21 kDa) and one heavy chain (41 kDa), which are connected by a disulfide bridge. The primary effect of activated protein C is to inactivate coagulation factors Va and VIIIa, which are necessary for the efficient generation of thrombin and the activation of factor X. Congenital protein C deficiency is most often transmitted as an autosomal dominant trait with varying degrees of penetrance [18]. Heterozygous protein C deficiency is an important independent risk factor for the development of deep vein thrombosis, [19] characterized by recurrent venous thrombosis. In this case, c.125C>A *PROC* mutation was identified in the proband, where CGT encoding Arg42 at the furin-like processing enzyme cleavage site was changed to AGT encoding Ser. This mutation abolishes the cleavage site required for production of mature protein C molecule [9]. In this mutation, the specificity of the processing protease would be shifted to a Lys-Ser bond to produce mutant protein C with loss of anticoagulant activity. Protein C Osaka 10 showed normal amidolytic activity and a normal protein C antigen level; however, it had an anticoagulant activity of only about 50% compared to that of the wild-type protein. This mutation is characterized as type 2 protein C deficiency. In this case, protein C antigen of the patient and her younger brother was decreased by administration of warfarin (Table 2). 

In a study of 173 patients with deep vein thrombosis, 55 (32%) were found to carry mutations in genes encoding protein S, protein C, and antithrombin [11]. However, deficiency of protein C occurs in a variety of other conditions, such as severe liver disorders, the nephrotic syndrome, acute respiratory distress, and postoperative states [20], suggesting the importance of extending investigations of *PROC* particularly to patients suffering from multiple thrombi. The frequency of heterozygous protein C deficiency may be as high as 1/200 to 1/500 in healthy adults; however, those affected do not exhibit thrombotic manifestations [21, 22]. This study also indicates that other unidentified factors may play an important role in the clinical signs of thrombosis formation. 

The patient, her father, and younger brother described here express Protein C Osaka 10. However, her father and younger brother showed no detectable thrombi. The implementation of primary prophylaxis with aspirin, heparin, or warfarin is often considered in known familial cases. Anticoagulant prophylaxis is given to all patients who develop a venous clot regardless of the underlying cause. Patients with protein C deficiency are at increased risk of recurrent venous thromboembolic events. Long term anticoagulation therapy using warfarin may be considered in these patients [23]. Therefore, we believe that familial screening for *PROC* mutations could avoid new thrombotic complications as well as improving the long term prognosis of the patients with Budd-Chiari syndrome caused by protein C deficiency. 

We suggest a hypothesis to account for the absence of detectable thrombosis in our patient's family members, these individuals harbored the same mutation as our patient with a concomitant decrease in protein C activity. The hypothesis maintains that elevated levels of total cholesterol may be a risk factor for venous thromboembolism (VTE). Studies on the association between lipid profile and VTE are inconsistent, with some reporting that total cholesterol is the risk factor [24], while others report that lipid levels do not influence the risk of VTE; however, the levels of LDL are significantly associated with unprovoked VTE as revealed by univariate analysis [25]. Further, statins can prevent VTE [26]. The levels of total cholesterol and LDL cholesterol in our patient (total cholesterol, 299 mg/dL, LDL cholesterol, 195 mg/dL) were higher than those of her younger brother (total cholesterol, 234 mg/dL, LDL cholesterol; 161 mg/dL) and father (total cholesterol, 200 mg/dL, LDL cholesterol, 99 mg/dL). Moreover, we noted no recurrence of thrombosis after statins were administered to our patient. 

In summary, we describe here a case of Budd-Chiari syndrome with multiple venous thrombi caused by an Arg42Ser mutation in *PROC* and in two of three of her family members without detectable thrombosis. Thus, the patient's brother and father did not experience thrombotic events, even though they harbored the same mutation with a concomitant decrease in protein C activity. Further, the difference between the patient's complicated thrombosis and family members with no thrombosis, even though they all harbor the same *PROC* mutation, may be associated with lipid levels. Therefore, the contribution of this mutation to thrombogenicity remains to be determined.

## Figures and Tables

**Table 1:** Laboratory test values of the proband upon admission.

Complete blood count	
White blood cell count	6,600/*μ*L
Red blood cell count	396 × 10^4^/*μ*L
Hemoglobin	12.4 g/dL
Hematocrit	37.6%
Platelets	16.0 × 10^4^/*μ*L
Blood biochemistry	
Aspartate aminotransferase	29 IU/L
Alanine aminotransferase	46 IU/L
Alkaline phosphatase	175 IU/L
Lactate dehydrogenase	190 IU/L
*γ*-GTP	39 IU/L
Total bilirubin	0.8 mg/dL
Creatine phosphokinase	79 IU/L
Total cholesterol	299 mg/dL
Triglyceride	104 mg/dL
HDL-cholesterol	72 mg/dL
LDL-cholesterol	195 mg/dL
Sodium	143 mEq/L
Chloride	108 mEq/L
Potassium	3.9 mEq/L
Uric acid	3.1 mg/dL
Blood urea nitrogen	8 mg/dL
Creatinine	0.57 mg/dL
Total protein	5.1 g/dL
Albumin	3.5 g/dL
C-reactive protein	0.20 mg/dL
Hemoglobin A1c	5.6%
Thyroid-stimulating hormone	1.72 *μ*IU/mL
Thyroxine, free (fT4)	0.8 pg/mL
Thyroxine, free (fT3)	2.0 pg/mL
BNP	21.1 pg/mL
Coagulation	
APTT	32.5 s
PT-INR	1.06
D-dimer	1.40 *μ*g/mL
Immunochemistry	
Immunoglobulin G	320 mg/dL
Immunoglobulin A	71 mg/dL
Immunoglobulin M	114 mg/dL
Immunoglobulin E	17.6 IU/mL
Complement titer (CH50)	52.8 U/mL
Complement C3	111 mg/dL
Complement C4	37 mg/dL
Antinuclear antibody	<40
Lupus anticoagulant	(−)
HBs antigen	(−)
HCV-antibody	(−)
<ELISA>	
MPO-ANCA	93 EU
PR3-ANCA	<10 EU
Urinalysis	
pH	7.0
Specific gravity	1.010
Glucose	(−)
Protein	(−)
Cast	(−)
Erythrocytes	1–5/HPF
Leukocytes	1–5/HPF

Abbreviations: *γ*-GTP: gamma-glutamyl transpeptidase; HDL cholesterol: high-density lipoprotein-cholesterol; LDL cholesterol: low-density lipoprotein cholesterol; PR3-ANCA: proteinase-3 antineutrophil cytoplasmic antibody; MPO-ANCA: myeloperoxidase antineutrophil cytoplasmic antibody; APTT: activated partial thromboplastin time; BNP: B-type natriuretic peptide; PT-INR: prothrombin time-international normalized ratio.

**Table 2:** Analysis of clotting factors.

	Age	Gender	Arg42Ser mutation	Protein C anticoagulant activity (%)	Protein C antigen (%)	Protein S activity (%)	D-dimer (mg/mL)
Patient	35	Female	+	44	46*	85	1.4
Younger brother	31	Male	+	62	48*	118	0.22
Father	60	Male	+	61	128	147	0.26
Mother	61	Female	−	105	97	98	0.22

*Administration of warfarin.
